# Constructing a tumor immune microenvironment-driven prognostic model in acute myeloid leukemia using bioinformatics and validation data

**DOI:** 10.1038/s41598-025-03557-9

**Published:** 2025-07-18

**Authors:** Amir Abbas Navidinia, Ali Keshavarz, Bentol Hoda Kuhestani Dehaghi, Reza Khayami, Najibe Karami, Vahid Amiri, Mehdi Allahbakhshian Farsani

**Affiliations:** 1 Department of Hematology and Blood Banking, School of Allied Medical Sciences, Shahid Beheshti University of Medical Sciences, Tehran, Iran; 2 Department of Medical Genetics and Molecular Medicine, Faculty of Medicine, Mashhad University of Medical Sciences, Mashhad, Iran; 3 Hematology-Oncology and Stem Cell Transplantation Research Center, Tehran University of Medical Sciences, Tehran, Iran

**Keywords:** Acute myeloid leukemia, Tumor immune microenvironment, Prognostic model, TCGA, Immune-Related differentially expressed genes, Macrophage, Cancer microenvironment, Computational biology and bioinformatics

## Abstract

The tumor immune microenvironment (TIME) is a critical determinant of prognosis in acute myeloid leukemia (AML). This study aimed to develop a prognostic model based on immune-related hub differentially expressed genes (hub-DEGs) to refine risk stratification and identify therapeutic targets. Transcriptomic and clinical data from 149 TCGA-AML patients were analyzed using ESTIMATE and xCell algorithms to infer immune scores. Differentially expressed genes (DEGs) between high/low immune score groups were identified, followed by functional enrichment, protein-protein interaction (PPI) network analysis for selecting the hub-DEGs with the highest degree scores, and univariate Cox regression to pinpoint prognostic genes. External validation was performed on 562 GEO-AML patients. The final genes were selected by intersecting the prognostic DEGs and hub-DEGs. Next the immune prognostic model (IPM) was created using these genes. xCell and CIBERSORT algorithm were used to assess the correlation of IPM and different immune cells. Finally, Experimental validation of key genes (CD163, MRC1) was conducted via RT-PCR in 40 AML and 10 control samples. Immune scores correlated with FAB classification (ESTIMATE: *p-value* = 1.4e − 8; xCell: *p-value* = 3.7e − 9) and overall survival (ESTIMATE: v = 0.041). Analysis identified 680 immune-related DEGs enriched in immune response pathways. Intersection of prognostic DEGs (*n* = 34) and hub-DEGs (*n* = 30) yielded four genes (CD163, IL10, MRC1, FCGR2B). A risk score model stratified patients into high/low-risk groups with divergent survival (*p-value* = 0.00072). ROC analysis demonstrated predictive accuracy (AUC: 63.38–68.5% for 1–5-year survival). TIME analysis revealed associations between high-risk scores and immunosuppressive cell subsets, including Tregs and M2 macrophages. RT-qPCR confirmed elevated CD163 in AML (*p* < 0.001), while MRC1 showed no differential expression. This study establishes a TIME-centric prognostic model with clinical utility for risk stratification and therapeutic targeting in AML. Prospective validation and integration of advanced genomic technologies are warranted to refine its translational applicability.

**Supplementary Information:**

The online version contains supplementary material available at 10.1038/s41598-025-03557-9.

## Introduction

Acute myeloid leukemia (AML) represents a heterogeneous spectrum of conditions characterized by the unbridled proliferation and differentiation of myeloid cells^1^. Initial interventions for AML entail the administration of non-specific yet highly potent chemotherapy agents, such as cytarabine and anthracyclines. Subsequent to this phase, the ongoing management strategy revolves around an in-depth assessment of the patient’s prognosis landscape and inherent risk profile^2^. This personalized approach seeks to tailor therapeutic interventions, directing heightened therapeutic intensity towards individuals manifesting an elevated risk of disease progression, all the while striving assiduously to mitigate the burden of treatment-related toxicity in the case of patients deemed to be of lower risk^3^. Although, the majority of AML patients attain a state of remission after undergoing induction therapy, regrettably, relapse remains a prevalent occurrence, and with each instance of relapse, the prospects for sustained survival diminish progressively^4,5^. Notably, the estimated 5-year overall survival (OS) rate approximates 30%, with notable variations observed across different age groups; younger patients demonstrate a 5-year survival rate of around 50%, in contrast to their elderly patients aged over 60, who encounter a considerably diminished rate, falling below 10%^6^.

The tumor microenvironments (TME) comprises a complex network of stromal cells (e.g., fibroblasts, mesenchymal and endothelial cells), immune cells (B and T lymphocytes, natural killer (NK) cells, and tumor-associated macrophages (TAMs)), the extracellular matrix (ECM), and secreted factors, such as cytokines^7^. The TME, as a supportive niche for leukemic stem cells, have garnered growing attention due to their crucial involvement in the progression of cancer and their impact on the efficacy or resistance to therapeutic approaches^7–9^. For example, research findings have demonstrated that AML leukemic cells induce switching of M1 macrophages to M2 macrophages by producing arginase-II, resulting in increased levels of M2 macrophages in AML patients in comparison to control groups, and worse prognosis^10,11^. Furthermore, leukemic cells can exacerbate T cell apoptosis and foster regulatory T cell (Treg) populations by producing indoleamine 2,3-dioxygenase 1 (IDO1), ultimately leading to diminished relapse-free and overall survival rates in AML cases^12–14^. Given these intricate dynamics, there is a pressing need to delve comprehensively into the prognostic factors within the TME, with particular emphasis on immune cell interactions, to inform more effective clinical management strategies and therapeutic approaches for AML patients.

Bioinformatics analysis can provide a systematic and objective approach to identify immune cell subsets associated with clinical outcomes in AML patients. In recent years, several computational algorithms have been introduced that predict the infiltration of immune and stromal cells in TME using gene expression data. One such algorithm, xCell, is a new gene signature-based method developed using thousands of pure cell types from various sources. xCell provides a precise and sensitive method for detecting the enrichment of several cell types in an admixture, allowing for the detection of small variations in the enrichment of a specific cell type in the tumor microenvironment (TME) with high confidence^15^. ESTIMATE (Estimation of STromal and Immune cells in MAlignant Tumors tissues using Expression data) is another algorithm that uses single-sample gene set-enrichment analysis (ssGSEA) to estimate the purity of immune and stromal cells in TME^16^. According to previous studies, ESTIMATE has provided valuable insight into the study of hepatocellular carcinoma^17^, glioblastoma^18^, prostate cancer^19^, colon cancer^20^, breast cancer^21^, and gastric cancer^22^.

Despite the advances made in understanding the role of immune cells in AML prognosis, a comprehensive evaluation of immune cells in AML prognosis using bioinformatics analysis is still needed. The aim of this study is to perform a comprehensive evaluation of immune cells in AML prognosis using bioinformatics analysis. Our findings may provide new insights into the role of immune cells in AML prognosis and contribute to the development of personalized treatment strategies for AML patients.

## Materials and methods

### Data requisition

**Fig. 1:**
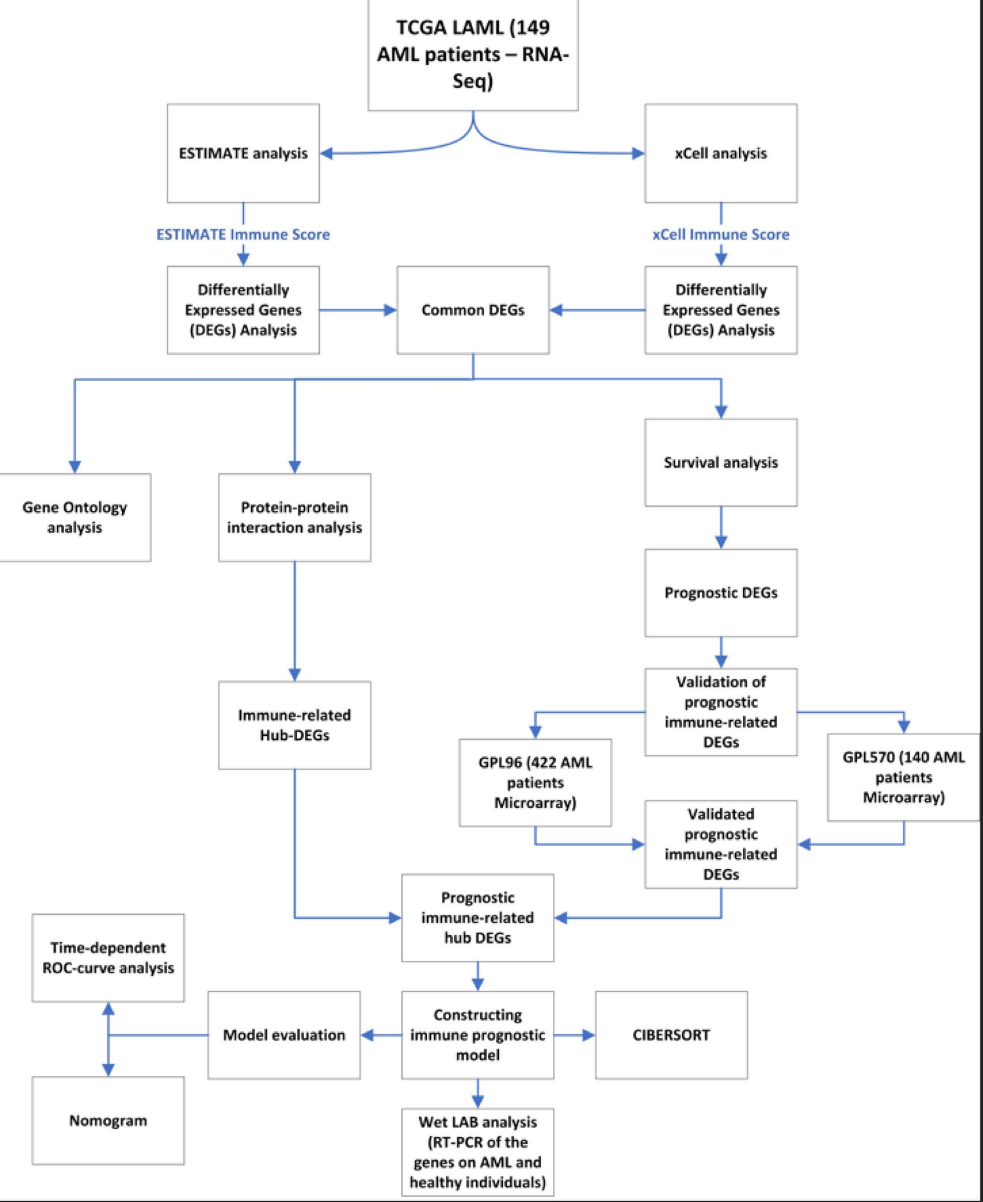
Schematic overview of the study workflow.

In this study, Level 3 RNA sequencing data, along with corresponding clinical information, were downloaded from the GDC database (https://portal.gdc.cancer.gov/repository) for a total of 173 newly diagnosed AML patients. Furthermore, mRNA expression array GSE37642 (GPL96 containing 422 adult AML patients, and GPL570 containing 140 adult AML patients) was obtained from the GEO database (https://www.ncbi.nlm.nih.gov/geo/). Within the TCGA cohort, 12 individuals were identified as having incomplete clinical records, and 12 patients had an overall survival of zero. Given that our primary objective was the development of a prognostic model, which relies on the availability of comprehensive clinical details, these 24 patients were subsequently excluded from the analytical process. As a result of this exclusion, the remaining cohort comprised 149 patients. The methodological approach employed in this research paper is illustrated by Fig. 1.

### Association of immune scores and clinical characteristic

In order to assess the purity of immune cells within TME of the 149 AML patients, an xCell and ESTIMATE analysis were conducted. Subsequently, based on the median values of xCell and ESTIMATE scores, the AML patients were split into low and high groups. Following this classification, an investigation into the correlation between the immune scores obtained from the xCell and ESTIMATE analyses and various clinical characteristics, including overall survival, FAB classification, and CALGB category, was undertaken. For the purpose of survival analysis, the “survival” package in the R programming environment was utilized.

### Immune-related differentially expressed genes

To ascertain immune-related differentially expressed genes (DEGs) between the high and low xCell groups, as well as high and low ESTIMATE groups, the “DESeq2” package was employed. DEGs were determined by considering genes with a |Fold Change| exceeding 1.5 and a false discovery rate (FDR) lower than 0.05. The visualization and representation of immune-related DEGs originating from both the xCell and ESTIMATE analyses were executed through the utilization of a combination of packages, including “pheatmap,” “plotPCA,” and “ggplot2.” These tools were utilized for the generation of informative heatmap plots, Principal Component Analysis (PCA) plots, and volcano plots, respectively. Subsequently, the immune-related genes commonly upregulated and downregulated were obtained and assembled for further investigation. A Venn diagram was generated using the “ggVennDiagram” package to visualize the shared gene sets.

### Protein-protein interaction and hub gene identification

STRING v.12 (https://string-db.org/) was used to investigate the immune related-DEGs’ interactions using a Protein-Protein Interaction (PPI) analysis and hub gene identification. The interactions retrieved from String were subsequently imported into Cytoscape software v.3.8.2 to construct the PPI network. For the purpose of hub gene identification, we employed the degree algorithm provided by CytoHubba, a Cytoscape plug-in known for its utility in network analysis. The immune-related hub DEGs, which represent central and highly connected nodes in the PPI network, were determined as the top 30 immune-related DEGs exhibiting the highest degree scores within the PPI network.

### Gene ontology and REACTOME pathway analysis

Gene Ontology (GO) and REACTOME analysis were performed on DEGs by using (DAVID, http://david.niaid.nih.gov), and *P* < 0.05 were considered statistically significant.

### Survival analysis and validation in GEO

In order to assess the prognostic value of immune related-DEGs, univariate Cox regression analysis was conducted through the utilization of the R package “survival” to examine the associations between the expression levels of DEGs and the overall survival of the patients. Immune related-DEGs with *P* < 0.05 were identified as prognostic genes. Subsequently, to validate the prognostic value of these immune related-DEGs, we performed univariate cox regression analysis on the GEO data containing 562 AML patients. Those immune-related DEGs within this dataset that exhibited a significance level of *P* < 0.05 were acknowledged as validated prognostic immune-related DEGs and were subsequently chosen for further analysis.

### Prognostic hub immune related-DEGs identification

To identify the prognostic hub immune related-DEGs, common genes between the validated prognostic immune-related DEGs and immune-related hub DEGs are selected.

### Immune prognostic model construction

To construct an Immune-Prognostic Model (IPM), the prognostic hub immune related-DEGs was randomly partitioned into training and test groups to validate the model’s accuracy. The train set was utilized to construct IPM, while both the testing set and the entire dataset were utilized to validate the predictive signature. The “glmnet” package was used to perform LASSO regression analysis (using the penalty parameter estimated by 10-fold cross-validation) to narrow the risk of overfitting. Risk score (RS), which statistically equals Σ (βi × Expi) (i = the number of prognostic hub genes), was calculated for every AML patient based on multivariate Cox regression analysis.

To assess the model’s accuracy comprehensively, an array of R packages including “survival,” “caret,” “glmnet,” “rms,” “survminer,” and “timeROC” were employed. These packages facilitated the execution of various analyses, including Kaplan-Meier (KM) analysis and the generation of ROC curves for 1, 3, and 5-year survival, across the training, testing, and entire patient datasets. Additionally, the calculation of the area under the curve (AUC) was carried out, providing a valuable measure of the model’s predictive performance for training, testing, and entire patient datasets. A higher AUC value indicated enhanced predictability of the IPM under the ROC curve. Subsequently, a nomogram model was established for forecasting survival years in AML patients by incorporating the risk score and various clinical features, such as age, FAB classification, and CALGB stage, utilizing the “rms” and “survival” packages. The Consistency Index (C-index) was then computed to assess the model’s accuracy and provide insights into its reliability and effectiveness.

### Immune cell profiling using CIBERSORT

The xCell (https://xcell.ucsf.edu/) and CIBERSORT (https://cibersortx.stanford.edu/) algorithms were applied to quantify the relative proportions of immune cell types. The xCell signatures were validated through extensive in silico simulations and cytometry-based immunophenotyping, and 32 distinct immune cell types were analyzed using xCell. CIBERSORT analysis was conducted with the LM22 gene signature matrix and 1000 permutations to ensure a robust estimation of immune infiltration across 22 immune cell types. Correlations between immune cell infiltration and high- and low-risk IPM groups were assessed using the t-test, with statistical significance set at *p* < 0.05. Additionally, correlations among immune cell types were visualized as heatmaps, generated using the “ggcor” R package.

### Patients and sample collection

Bone marrow (BM) and peripheral blood (PB) samples were collected from 40 newly diagnosed AML patients at Taleghani Hospital (Tehran, Iran), each confirmed by molecular, cytomorphological, and flow cytometric tests (Supplementary Table 1). In addition, 10 control samples (*n* = 7 BM and *n* = 3 PB) were obtained from individuals with low platelet counts but normal flow cytometric analysis. Written informed consent was obtained from all participants or their legal guardians. All methods were performed in accordance with the relevant guidelines and regulations, including the Declaration of Helsinki. The Ethics Committee of Shahid Beheshti University of Medical Sciences approved the study (approval code: IR.SBMU.RETECH.REC.1401.662).

### RNA analysis, cDNA synthesis, and RT-qPCR

Total RNA was extracted using QIAamp DNA Blood Kits (QIAGEN, Germany). Subsequently, cDNA synthesis was performed according to the protocol of RevertAid First Strand Kits (Thermo Scientific, USA). Primers were designed using Gene Runner version 3.05, and sequence analysis was conducted through Primer-BLAST (Table 1). Real-time PCR (RT-qPCR) was performed with SYBR™ Green Real-time PCR Master Mixes (Amplicon, Denmark). All experiments were conducted in duplicate. Gene expression fold changes were calculated using the Livak method ($\:{2}^{-\varDelta\:\varDelta\:CT}$), with ABL1 employed as the housekeeping gene^23^.

**Table 1:** Primer sequences and product lengths for target genes.

Gene	Primer type	Sequence	Length (bp)	Product length (bp)
ABL1	F	CTTCTTGGTGCGTGAGAGTGAG	22	115 bp
	R	GACGTAGAGCTTGCCATCAGAAG	23	
CD163	F	GCAGCACATGGGAGATTGTC	20	98 bp
	R	TTTGGGACTGGTTTCCTGAGC	21	
MRC1	F	CAGACACGATCCGACCCTTC	20	125 bp
	R	GTCTCCGCTTCATGCCATTG	20	

### Statistical analysis

R (version 4.4.2, Auckland, NZ, United States) software was used for all bioinformatic analyses. The Kruskal–Wallis test was employed to examine differences in variables among multiple groups and the t-test was used for comparing two groups. Moreover, the PCR data were analyzed using Graph Pad Prism (version 10). The Kolmogorov–Smirnov test was selected to assess the data distribution, and Mann–Whitney U was used to compare the relative expression of target genes. Differences were considered significant when *P* < 0.05.

## Results

### Estimate scores are associated with AML clinical parameters

We obtained the complete gene expression profiles and clinical data for 149 AML patients from TCGA. Of these patients, 80 (53.69%) were male, and 69 (46.31%) were female. The median age at initial pathological diagnosis was 57 years, ranging from 18 to 88 years. Based on the FAB classification, the patient cohort exhibited a range of subtypes. Notably, M1, M2, and M4 represented the most prevalent subtypes, with the percentages ranging from 19.3 to 20.5%. The M0 and M3 subtypes had lower prevalence, while M5, M6, and M7 subtypes were relatively rare. One patient (0.6%) remained unclassified under the FAB system. According to the CALGB classification, 31 patients were allocated to the favorable prognosis group, 90 were categorized within the intermediate/normal category, and 26 were designated as having a poor prognosis. Subsequently, in order to assess the immune scores of the patients, ESTIMATE and xCell algorithms were utilized. The ESTIMATE immune scores ranged from 1322 to 3959 (median: 2659), while the xCell immune scores ranged from 0.0041 to 0.4783 (median: 0.1065). Subsequently, based on the median scores on ESTIMATE and xCell, the patients were categorized into high immune score (HIS) and low immune score (LIS) groups.

**Fig. 2:**
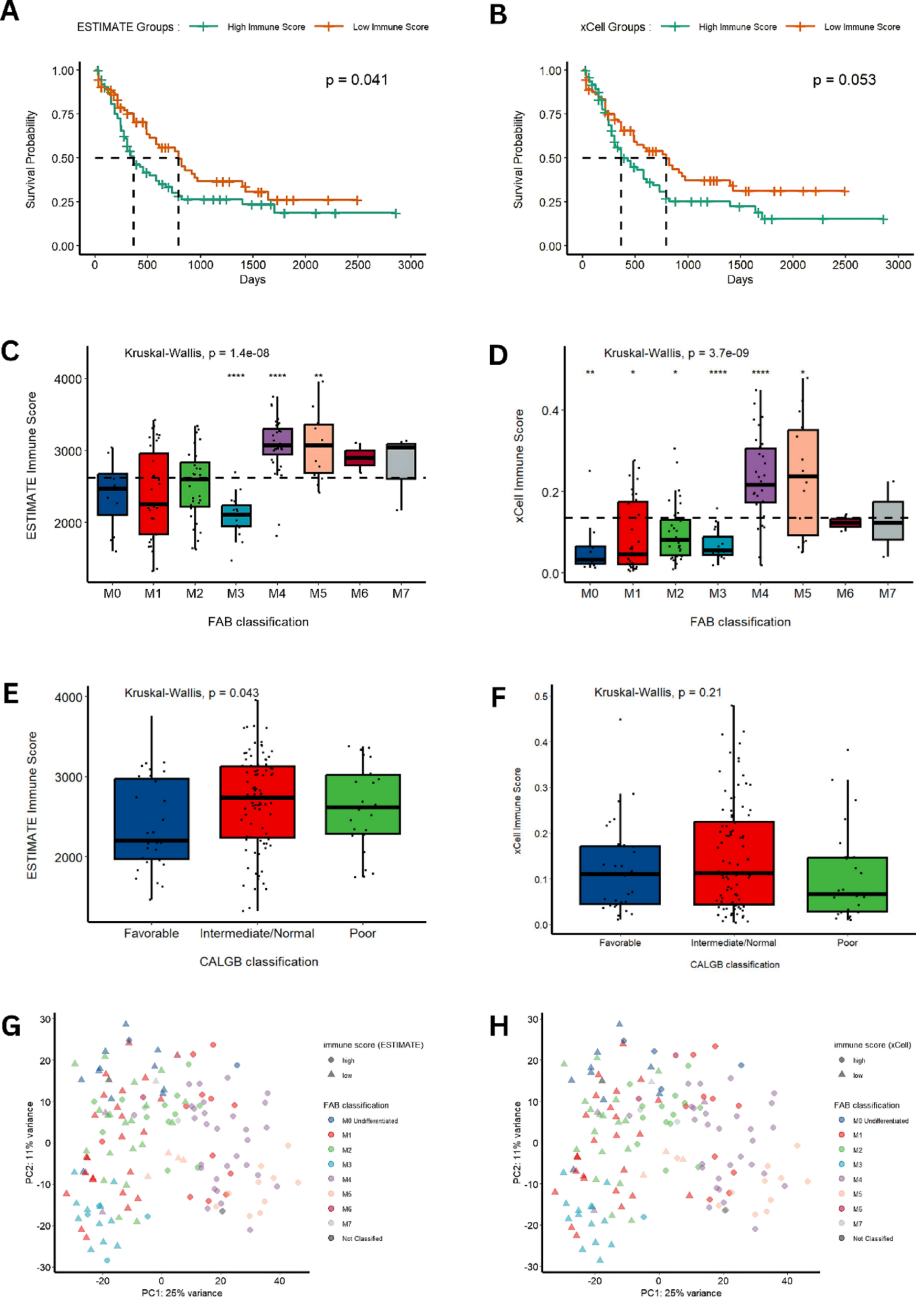
Comparative Analysis of ESTIMATE and xCell Immune Scores in AML. Kaplan–Meier survival curves in (**A**,**B**) highlight the difference in high vs. low immune scores calculated using ESTIMATE (**A**) and xCell (**B**). Similarly, the distribution of immune scores within the Cancer and Leukemia Group B (CALGB) categories is illustrated in (**C**) for ESTIMATE and (**D**) for xCell. The French-American-British (FAB) classification is compared across ESTIMATE immune scores in (**E**) and xCell immune scores in (**F**). Lastly, principal component analyses in (**G**) for ESTIMATE and (**H**) for xCell demonstrate the relationships among immune scores, CALGB categories, and FAB classification. Statistical significance levels are indicated as follows: *p < 0.05, **p < 0.01, ***p < 0.001, ****p < 0.0001.

To explore the potential association between immune scores and overall survival (OS), Kaplan–Meier analysis was performed. The results indicated that patients with ESTIMATE HIS exhibited a shorter median OS compared to those with ESTIMATE LIS (*p-value* = 0.041; Fig. 2A). However, there was no significant correlation between xCell immune scores and the patients’ OS (*p-value* = 0.53; Fig. 2B). Similarly, a statistically significant association emerged between the ESTIMATE immune scores and the CALGB category (*p-value* = 0.043; Fig. 2C), while the immune scores derived from the xCell algorithm did not exhibit a commensurate level of significance (*p-value* = 0.17; Fig. 2D).The immune scores originating from both algorithms demonstrated a striking statistical relevance concerning the FAB classification (ESTIMATE immune score: *p-value* = 1.4e-8, Fig. 2E; xCell immune score: *p-value* = 3.7e−9, Fig. 2F). Moreover, as a consequence of the PCA analysis, we elucidated the intricate connections between FAB classification and the ESTIMATE and xCell immune scores, unveiling complex data patterns and their relevance to both immune scores and FAB classification (Fig. 2G,H).

### Identification of Immune-related differentially expressed genes (DEGs)

**Fig. 3:**
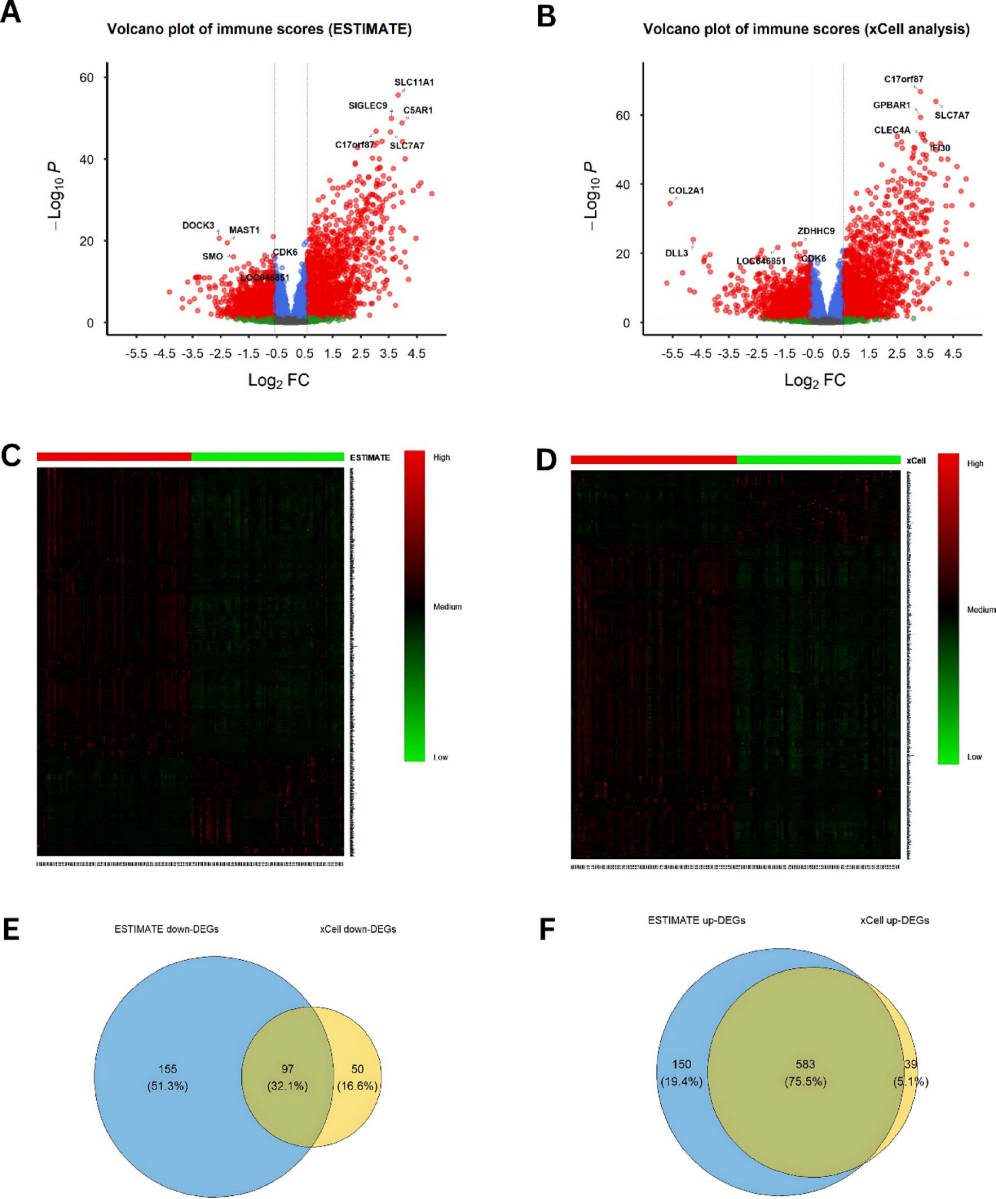
Identification of immune-related differentially expressed genes (DEGs). (**A**,**B**) Volcano plots show DEGs identified by ESTIMATE (**A**) and xCell (**B**); (**C**,**D**) Heatmaps display expression patterns of upregulated and downregulated DEGs in low vs. high immune score groups for ESTIMATE (**C**) and xCell (**D**); (**E**,**F**) Venn diagrams highlight the genes commonly downregulated (**E**) and upregulated (**F**) across both algorithms.

We identified the immune related-DEGs of both ESTIMATE and xCell algorithms by performing cut-off criteria of *adjusted p-value* = 0.05 and |log2 fold change| > 1.5. Based on ESTIMATE immune scores, 985 DEGs (733 upregulated genes and 252 downregulated genes were found, and based on xCell immune scores, 769 DEGs (622 upregulated genes and 147 downregulated genes) were found (Fig. 3A,B). The DEGs of the low versus high immune score groups are depicted heatmap (Fig. 3C,D). We identified 97 genes that exhibited a shared downregulation in expression and 583 genes that displayed a mutual upregulation, as illustrated in Fig. 3E,F, respectively. This amalgamation amounted to a total of 680 genes, which constituted a robust selection of immune-related DEGs derived from the ESTIMATE and xCell algorithms. Our subsequent investigation was primarily focused on this subset of common DEGs.

### Gene ontology and REACTOME pathway analysis

**Fig. 4:**
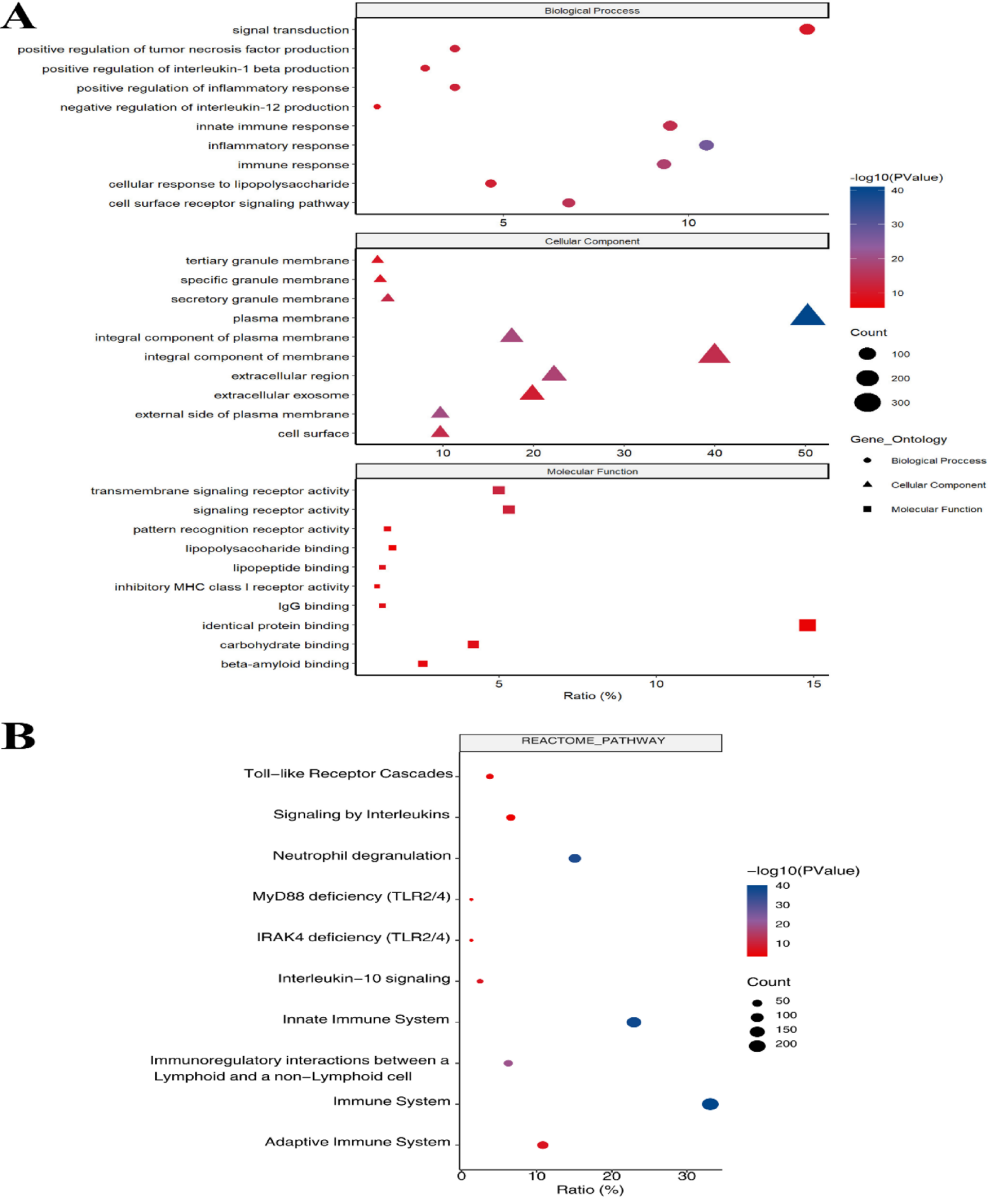
Gene ontology (GO) and REACTOME pathway analysis of immune-related DEGs. (**A**) The top 30 significantly enriched GO terms across biological process, molecular function, and cellular component sub-ontologies; (**B**) Interrelation analysis of REACTOME pathways for these DEGs.

Gene ontology (GO) and REACTOME pathway analyses were used to investigate the biological processes and pathways involved. Using the DAVID gene annotation tool, the DEGs were analyzed for three sub-ontologies, as shown in Fig. 4A: biological processes (BP), cellular components (CC), and molecular function (MF). Regarding BP, DEGs were most enriched in critical categories, including inflammatory response, immune response, cell surface receptor signaling pathway, innate immune response, and positive regulation of tumor necrosis factor production. Regarding molecular function, DEGs exhibited enrichment in various categories, including transmembrane signaling receptor activity, signaling receptor activity, lipopeptide binding, carbohydrate binding, and IgG binding. REACTOME pathway analysis revealed that the top pathways related to DEGs were the immune System, neutrophil degranulation, innate immune System, immunoregulatory interactions between a lymphoid and a non-lymphoid cell, Interleukin-10 signaling, toll-like receptor cascades, and cytokine signaling in the immune system (Fig. 4B).

### Selecting prognostic immune-related hub-DEGs

**Fig. 5:**
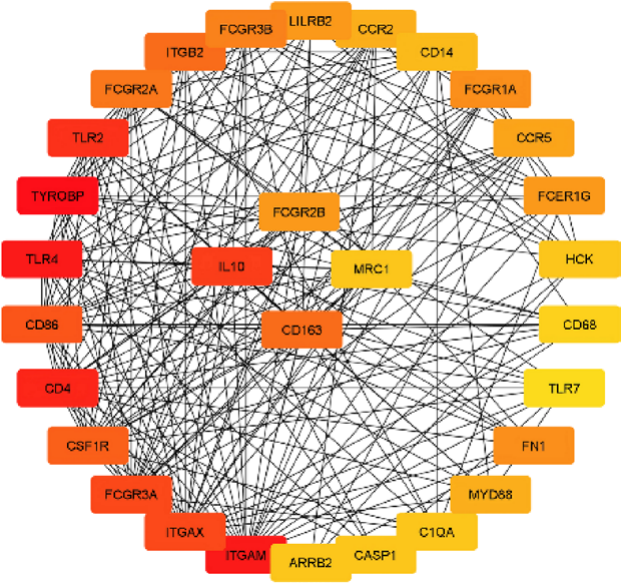
Top 30 Hub DEGs in the PPI network. A subnetwork of the 30 DEGs with the highest degree centrality scores. Nodes are colored from red (higher degree) to yellow (lower degree).

To identify prognostic immune-related hub-differentially expressed genes (DEGs) from a pool of 680 DEGs, we initiated the process by establishing a Protein-Protein Interaction (PPI) network. This network comprised 652 nodes and 5015 edges (Supplementary Fig. 1). Employing degree centrality analysis from the cytoHubba plugin, we selected the top 30 DEGs with the highest degree scores and designated as *immune-related hub DEGs* (Fig. 5, Supplementary Table 2).

Subsequently, a univariate Cox regression analysis was performed to identify prognostic DEGs. Among the 680 immune-related DEGs, 173 exhibited significant prognostic value in the TCGA dataset. For external validation, two GEO datasets, comprising 114 and 417 AML patients with complete clinical information, respectively, were analyzed using univariate Cox regression. Of the 173 prognostic DEGs, 34 were validated in GEO datasets and were thus designated as prognostic immune-related DEGs (Supplementary Table 3).

In the final stage, the prognostic immune-related hub DEGs were determined by identifying the intersection of validated prognostic DEGs and hub-DEGs. This process led to the identification of four genes: CD163, IL10, MRC1, and FCGR2B (Supplementary Fig. 2).

### Constructing prognostic model based on immune-related hub-DEGs

In order to constructing prognostic model based on prognostic immune-related hub-DEGs, we performed multi-cox proportional hazard test. Expression levels of four signature DEGs and corresponding coefficients derived from the Cox regression model were used to calculate the risk score for each patient as follows: risk score = CD163 expression × 0.6273871 + IL10 expression× 0.45065 + MRC1 expression ×2.186315 + FCGR2B expression × 1.327702.

**Fig. 6:**
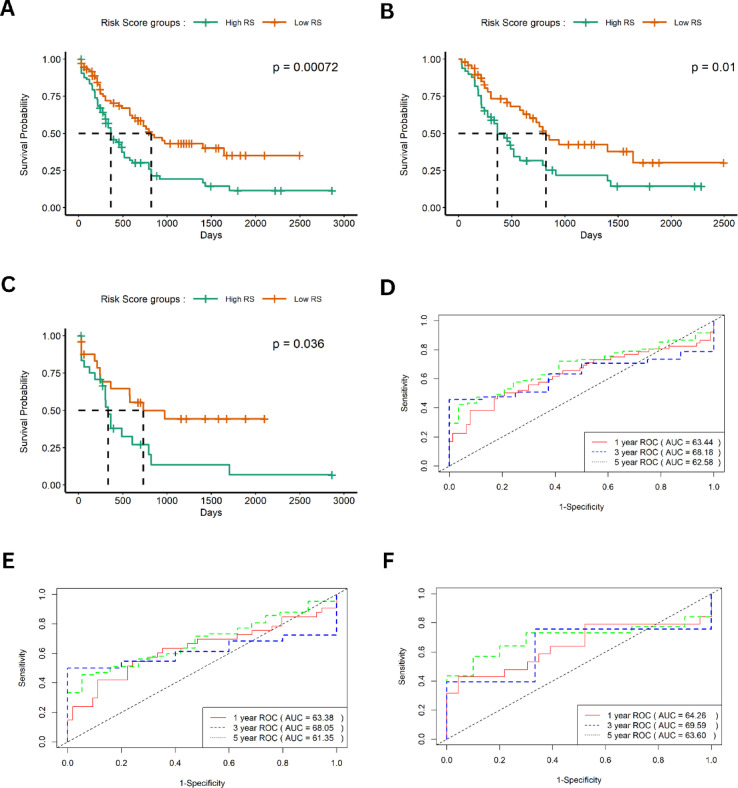
Construction and evaluation of the immune prognostic model. (**A**,**C**,**E**) Kaplan–Meier survival curves comparing high- vs. low-risk score (RS) groups in the entire cohort (**A**), training subset (**C**), and test subset (**E**). (**B**,**D**,**F**) Receiver operating characteristic (ROC) curves evaluating model performance in the entire cohort (**B**), training subset (**D**), and test subset (**F**).

The Kaplan–Meier survival analysis revealed that the high-risk group had a significantly lower survival rate compared to the low-risk group in the entire data (*p-value* = 0.00072), the training set (*p-value* = 0.01), and the test set (*p-value* = 0.036) (Fig. 6A,C,E). The receiver operating characteristic (ROC) curve was constructed to test the model’s accuracy in test, train, and entire data (Fig. 6B,D,F). The area under the curve (AUC) of 1, 3, and 5-year survival for entire data were 63.38%, 68.5%, and 61.35%, respectively, which indicate the robust predictive power of immune prognostic model across different timeframes.

### Nomogram model construction

**Fig. 7:**
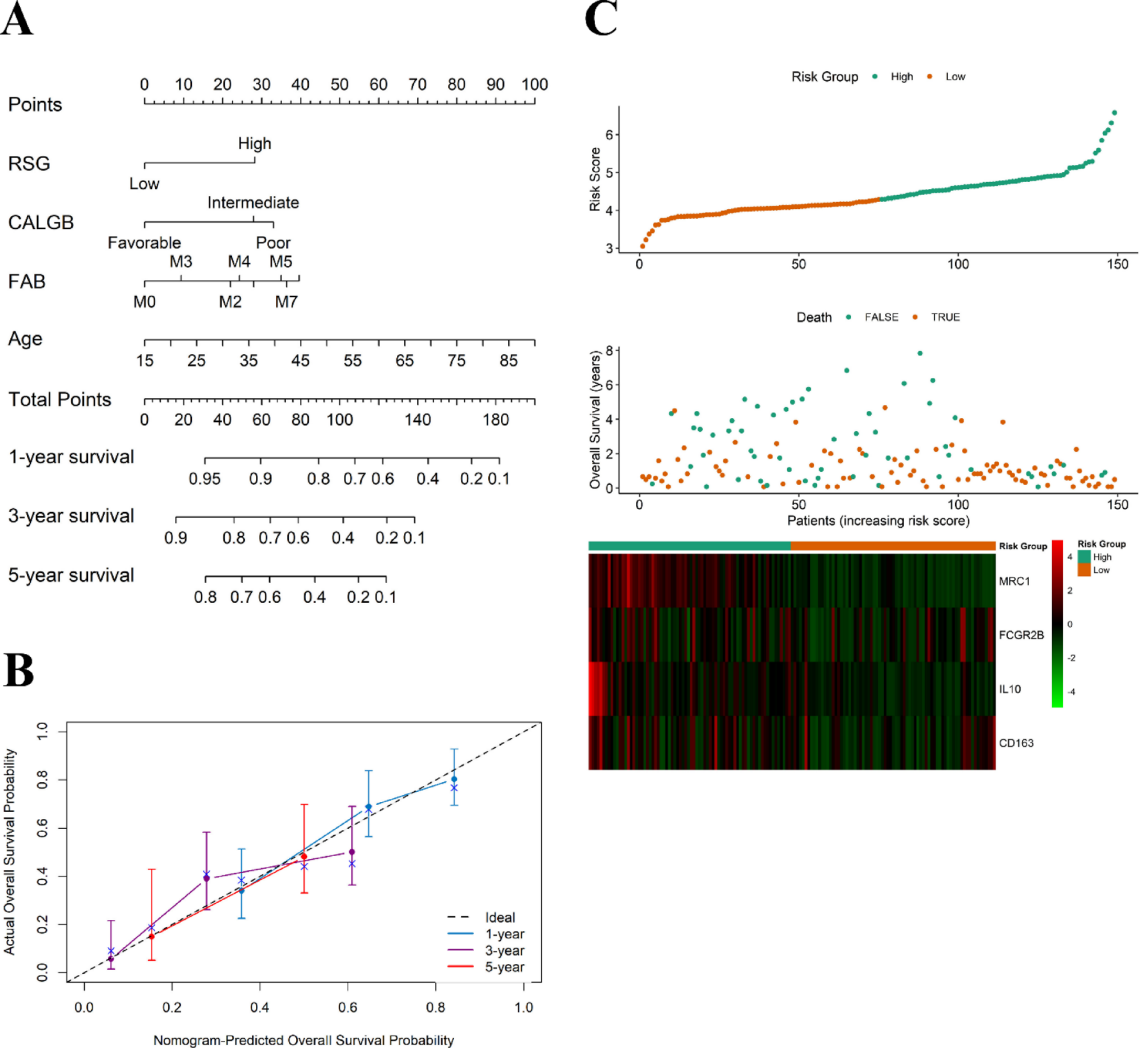
Nomogram, concordance index, and gene expression heat map. (**A**) Nomogram integrating the immune prognostic model (risk score, RS), patient age, French-American-British (FAB) classification, and Cancer and Leukemia Group B (CALGB) category to estimate 1-, 3-, and 5-year survival probabilities. (**B**) Concordance index (C-index) assessing the model’s discriminative power. (**C**) Heat map of the four signature genes (CD163, IL10, MRC1, FCGR2B) between high- and low-RS groups.

We established and validated a predictive nomogram tailored for predicting outcomes in AML patients. This nomogram, presented in Fig. 7A, integrates our microenvironment prognostic model, patient age, FAB classification, and CALGB category, providing risk assessments for patients at 1, 3, and 5-year intervals. Its development aimed to facilitate personalized risk evaluation and inform clinical decision-making. To gauge its performance in distinguishing patients who experienced the targeted clinical event from those who did, we employed the concordance index (C-index) (Fig. 7B). The expression patterns of the genes were further visualized in a heatmap comparing high- and low-RS groups (Fig. 7C).

### Correlation of the immune prognostic model with tumor immune microenvironment of AML

**Fig. 8:**
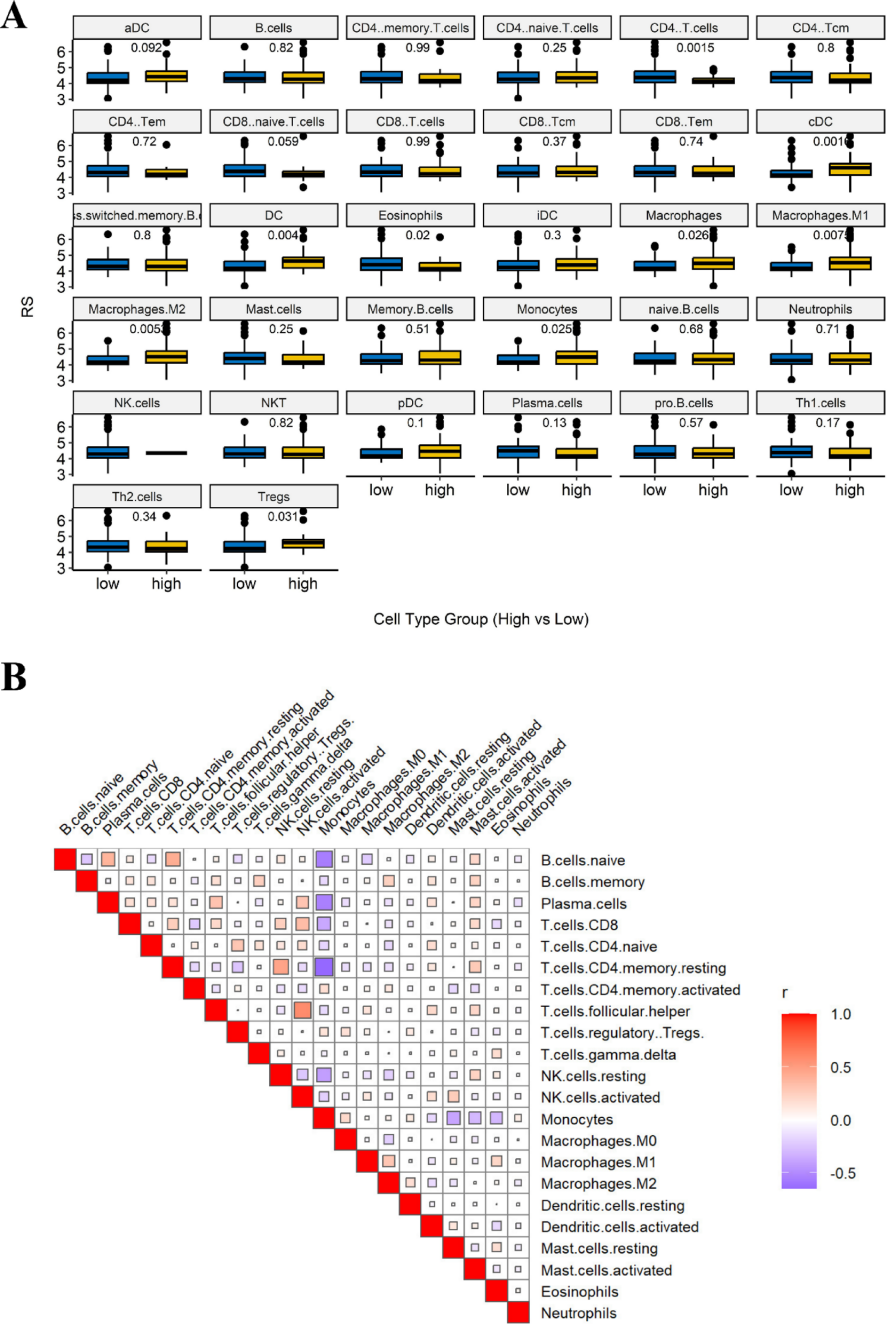
Correlation of the immune prognostic model with AML tumor immune microenvironment using CIBERSORT. (**A**) CIBERSORT-based infiltration levels of 22 immune cell types in high- vs. low-RS groups. (**B**) Correlation matrix of immune cell infiltration patterns derived from CIBERSORT.

We investigated the association between our immune prognostic model and the tumor immune microenvironment (TIME) in AML using CIBERSORT and xCell. CIBERSORT analysis of 22 unique TIME cell populations revealed that out IPM is significantly associated with the infiltration levels of activated CD4 + memory T cells (*p-value* = 0.029), naïve CD4 + T cells (*p-value* = 0.0067), regulatory T cells (Tregs; *p-value* = 0.03), and monocytes (*p-value* = 0.042) (Fig. 8A). To further explore interactions among TIME components, we performed a correlation analysis of immune cell infiltration patterns using CIBERSORT (Fig. 8B).

**Fig. 9:**
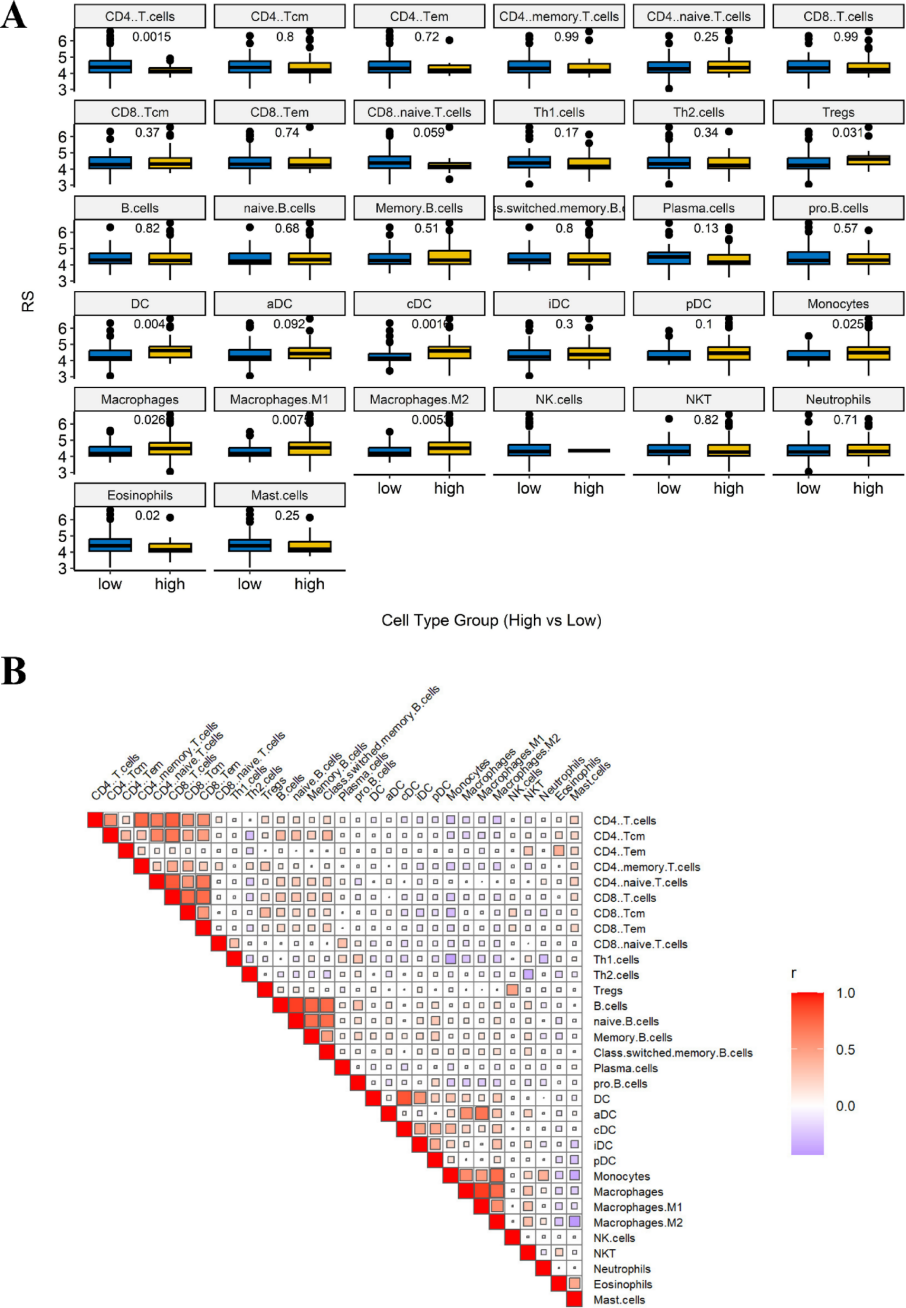
Correlation of the immune prognostic model with AML tumor immune microenvironment using xCell. (**A**) xCell-based infiltration levels of 32 immune cell types in high- vs. low-RS groups. (**B**) Correlation matrix of immune cell infiltration patterns derived from xCell.

Furthermore, xCell analysis of 32 distinct TIME cell populations identified nine immune cell types significantly associated with our prognostic model, including CD4 + T cells (v = 0.0015), Tregs (*p-value* = 0.031), dendritic cells (DCs; *p-value* = 0.004), conventional dendritic cells (cDCs; *p-value* = 0.0016), monocytes (*p-value* = 0.025), macrophages (*p-value* = 0.026), M1 macrophages (*p-value* = 0.0075), total macrophage populations (*p-value* = 0.0053), and eosinophils (*p-value* = 0.02) (Fig. 9A). Additionally, we conducted a correlation analysis of immune cell infiltration patterns using xCell, further illustrating TIME interactions (Fig. 9B).

### The expression of CD163 and MRC1 in our local cohort of AML patients and healthy individuals

**Fig. 10:**
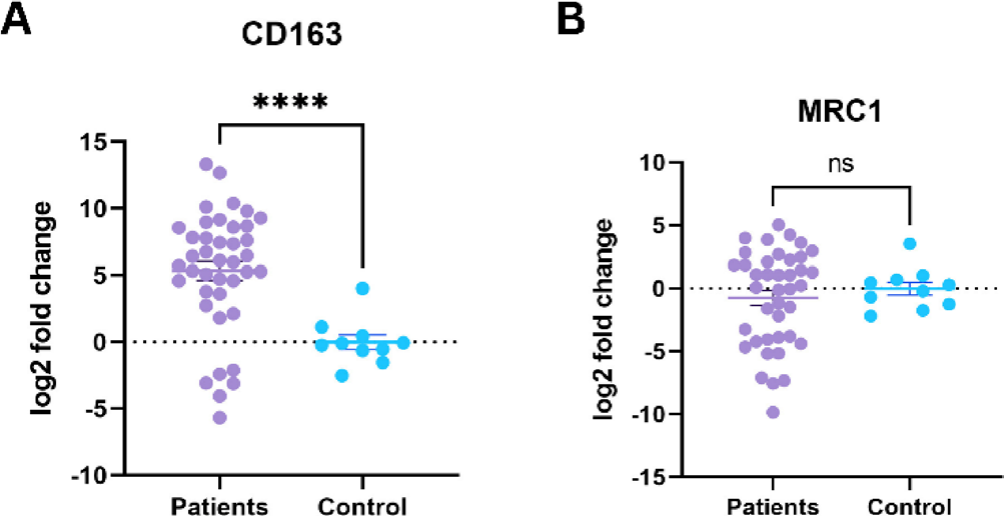
Expression of CD163 and MRC1 in AML vs. healthy controls.

To further validate the bioinformatic findings, we measured the expression levels of two candidate prognostic genes, CD163 and MRC1, in blood and bone marrow samples collected from our local cohort of AML patients and healthy controls. Real-time PCR analyses revealed that CD163 mRNA expression was significantly higher in AML patients than in controls (*p* < 0.001), whereas MRC1 expression did not differ significantly between the two groups (Fig. 10).

## Discussion

AML prognosis is influenced by several factors, including genetic abnormalities; however, in recent years, much attention has been paid to the TME, as the bone marrow is the site of leukemia initiation and development, and the stromal and immune cells within the TME play a crucial role in the proliferation, progression, drug resistance and survival of leukemic cells^24,25^. This study systematically delineated the prognostic relevance of immune-related gene signatures within the TME, culminating in the development of a robust risk stratification model. By integrating transcriptomic profiling, computational deconvolution algorithms, and clinical validation, our analysis identified four hub genes—IL10, MRC1, CD163, and FCGR2B—as central mediators of AML prognosis. These findings highlight the utility of immune-centric bioinformatics frameworks in elucidating molecular drivers of disease heterogeneity and advancing precision oncology paradigms.

Initially, the IS of AML patients was computed using ESTIMATE and xCell algorithms, revealing a significant association of IS with clinical characteristics such as overall survival, FAB classification, and CALGB category, thereby emphasizing the potential utility of immune profiling in prognostication and therapeutic stratification for AML patients. Subsequently, we identified commonly upregulated and downregulated DEGs between the two algorithms, selecting 680 genes associated with immune scores. Functional enrichment analysis of these DEGs demonstrated their involvement primarily in inflammatory response, immune response, cell surface receptor signaling pathway, innate immune response, and positive regulation of tumor necrosis factor production. Subsequently, a PPI network was constructed for the 680 DEGs obtained from the two algorithms, and the top 30 hub genes were selected based on degree scores. Moreover, an analysis of the relationship between DEGs and overall patient survival was conducted, identifying 173 DEGs associated with AML patient survival out of the 680 identified DEGs. Importantly, we validated the prognostic value of candidate DEGs using data from 562 patients from two GEO datasets. Ultimately, 34 DEGs were confirmed as prognostic candidate genes.

In the subsequent phase, the intersection of validated prognostic genes and hub genes led to the identification of four critical genes: IL10, MRC1, CD163, and FCGR2B. These genes were instrumental in constructing the IPM. The IPM exhibited good accuracy and prognostic efficacy. Through the application of Kaplan–Meier survival analysis, the model proficiently distinguished between high-risk and low-risk patient cohorts, thereby demonstrating its capability to accurately stratify patients according to their prognostic risk. Furthermore, the generation of ROC curves for 1, 3, and 5-year survival intervals corroborated the model’s robustness, affirming its reliability in predicting long-term survival outcomes. The model’s significant correlations with pivotal clinical parameters—such as patient age, FAB classification, and cytogenetic status—highlighted its clinical relevance. These correlations substantiated the IPM’s utility in clinical settings, facilitating precise risk stratification and informing the development of personalized therapeutic strategies for patients with AML.

MRC1 (Mannose Receptor C Type 1, CD206, located at 10p12.33) encodes a member of the C-type lectin receptor family, known as the human mannose receptor (MR). MRC1/CD206 is predominantly expressed on the surface of M2 immunosuppressive macrophages and TAMs, plays a significant role in fostering an immunosuppressive milieu conducive to tumorigenesis^26,27^. Kramer et al. reported that the protein MRC1 are highly expressed on AML blasts from some patients but not on CD34 stem/progenitor cells^28^. They also suggested that MRC1/CD206 could be a potential effective target for directing AML leukemic cells^28^. Furthermore, in line with our findings, other studies showed that MRC1 is upregulated in AML compared to healthy volunteers and serves as a potential biomarker for AML, laying the groundwork for further development of targeted therapies aimed at suppressing excessive MRC1 expression in AML^29,30^.

CD163, a scavenger receptor found on macrophages, encoded by the gene located at q12.213, produces a protein involved in the clearance of cysteine-rich scavenger receptors (SRCR) and serves as a marker for TAMs exhibiting pro-tumoral characteristics^31^. CD163^+^-TAMs are implicated in fostering an immunosuppressive microenvironment^31,32^. Moreover, CD163 is implicated in the pathogenesis of various cancers, including chronic lymphocytic leukemia, multiple myeloma, meningioma, Hodgkin’s lymphoma, and colorectal cancer^33–37^. In our investigation, we found a significant association between CD163 expression and AML prognosis, consistent with prior research^29,38^. Moreover, we observed a significant association between CD163 expression in AML and healthy individuals, indicating that CD163 is a diagnostic marker for AML. Thus, taken together, CD163 holds promise as a prognostic and diagnostic factor in AML and warrants further investigation regarding CD163 targeting and targeted drug development.

IL-10 holds a pivotal role in AML, orchestrating immunosuppressive functions, promoting AML cell survival and proliferation, and facilitating adhesion to BM-MSCs. Importantly, IL-10 can originate from both AML leukemic cells and the TME cells such as TAMs, DCs, and MSCs^39–41^. IL-10’s impact extends beyond mere support for tumor cell survival; it has been implicated in promoting the stemness of leukemia stem cells via IL10/IL10R/PI3K/AKT signaling pathway, thereby potentially perpetuating disease relapse and resistance to therapy^42^. Our study unveiled a strong correlation between elevated IL-10 levels in AML patients and unfavorable prognosis. Additionally, we found that this interleukin is more correlated with complex karyotype.

FCGR2B, also known as Fc Gamma Receptor IIb or CD32b, situated at 1q23.3, is increasingly recognized as a pivotal factor influencing the progression and treatment responses in leukemia and lymphoma cases^43,44^. This receptor is expressed across a spectrum of immune cell types, including DCs, TAMs, B-lymphocytes, activated neutrophils, mast cells, and basophils^45–47^. Research has elucidated that FCGR2B expression on mononuclear phagocytes is upregulated within the hypoxic TME, leading to a compromised phagocytic capability of these cells against cancer cells targeted by therapeutic antibodies^48,49^. Consequently, this phenomenon contributes to resistance against therapeutic antibodies, suggesting that interventions targeting FcγRIIB signaling hold promise for overcoming therapeutic resistance, either as monotherapy or in combination with other treatment treatments^48^. Moreover, intriguing findings by Parting et al. underscore the potential of targeting downstream signaling of FcγRIIb. Their study demonstrated that a combination of ibrutinib (a Bruton’s tyrosine kinase inhibitor) and standard TKI therapy significantly enhances apoptosis in quiescent chronic myeloid leukemia (CML) stem cells, thereby facilitating the eradication of leukemic stem cells (LSCs)^50^. Our investigation unveiled a significant elevation in FCGR2B expression levels in AML patients, which was closely correlated with unfavorable prognosis and reduced overall survival. These findings underscore the potential of FCGR2B as both a prognostic marker and a therapeutic target in AML.

We subsequently revealed a significant correlation between the IPM and various immune cell types, including CD4 + memory T cells, naïve CD4 + T cells, regulatory T cells, monocytes, dendritic cells, conventional dendritic cells, macrophages, M1 macrophages, M2 macrophages, and eosinophils. These findings highlight the diverse roles of IL10, MRC1, CD163, and FCGR2B in shaping the tumor immune microenvironment and further highlight their prognostic significance in AML. A growing body of evidence highlights the critical role of LAMs in AML progression and prognosis. For instance, a previous study indicated that a decrease in M1 LAMs and a prevalent presence of immunosuppressive M2 LAMs in both newly diagnosed and relapsed AML patients. These immunosuppressive M2 LAMs are characterized by the expression of CD163 and MRC1, with a considerable subset of CD206 + LAMs co-expressing CD163^51^. Moreover, another study has shown that the presence of CD163+/CD206 + LAMs identifies patients with the poorest prognosis^52^. Weinhäuser et al. found that the poor OS prediction associated with high CD163/CD206 mRNA expression is not necessarily driven by intrinsic AML biology but could be attributed to the presence of an AML tumor supportive niche^39,52,53^. Our molecular analysis revealed that while CD163 expression was significantly elevated in AML patients compared to healthy individuals, MRC1 expression remained unchanged. This seems that MRC1 has diagnostic impact but CD163 has both diagnostic and prognostic impact.

This investigation is subject to several constraints requiring resolution in subsequent studies. The prognostic algorithm was constructed using retrospectively acquired datasets, necessitating prospective validation to establish its clinical utility. Furthermore, experimental validation was restricted to assessing MRC1 and CD163 expression via RT-PCR in AML cohorts and healthy comparators. To comprehensively evaluate the model’s biological relevance, subsequent investigations should prioritize inclusion of FCGR2B and IL10 expression profiles across these populations. Although multiplex PCR remains the benchmark methodology for confirming pivotal results, emerging high-resolution techniques—including single-cell transcriptomic profiling, droplet digital PCR, and next-generation genomic platforms—present opportunities for substantial model refinement. Integration of such technologies could elucidate tumor microenvironment heterogeneity at cellular resolution, clarify molecular interactions driving disease progression, and enhance diagnostic precision through multidimensional biomarker characterization. These advancements may further inform the development of stratified therapeutic interventions tailored to distinct AML subtypes. Notwithstanding these limitations, the predictive framework outlined herein demonstrates significant potential as a platform for therapeutic target discovery and advancement of precision medicine paradigms in AML management. Systematic incorporation of multimodal molecular data and longitudinal clinical validation will be critical to translating these computational insights into robust diagnostic workflows and mechanistically grounded treatment strategies.

## Conclusion

In conclusion, this study establishes a TIME (Tumor Immune Microenvironment)-driven prognostic model with potential to inform therapeutic decision-making and target discovery in AML. While retrospective design and technical constraints necessitate cautious interpretation, the framework provides a scaffold for integrating multi-omics data and advancing mechanism-based therapies. Systematic validation through longitudinal studies and translational pipelines will be imperative to harness these insights for improving patient outcomes in this molecularly heterogeneous malignancy.

## Electronic supplementary material

Below is the link to the electronic supplementary material.

Supplementary Material 1

Supplementary Material 2

Supplementary Material 3

## Data Availability

The datasets generated and/or analyzed during the current study are available in the GDC repository (https://portal.gdc.cancer.gov/) and the GEO repository under the accession GSE37642 (https://www.ncbi.nlm.nih.gov/geo/).
