# Supplementary figures and images for: Constructing a tumor immune microenvironment-driven prognostic model in acute myeloid leukemia using bioinformatics and validation data
Source: Sci Rep. 2025 Jul 18;15:26123. doi: 10.1038/s41598-025-03557-9 (PMC12274339; doi:10.1038/s41598-025-03557-9)

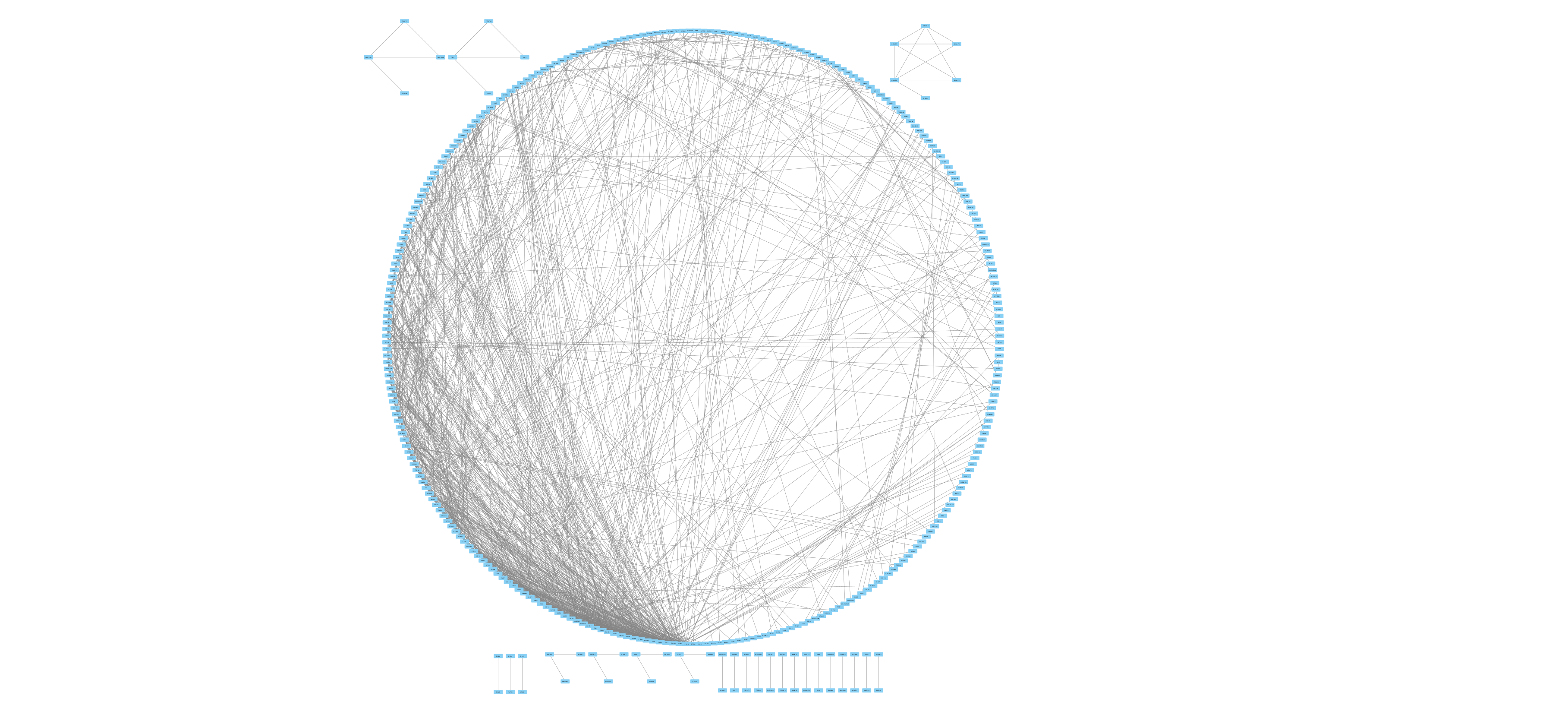

Supplement: Supplementary file 1 — Supplementary Material 1 [file 41598_2025_3557_MOESM1_ESM.png]

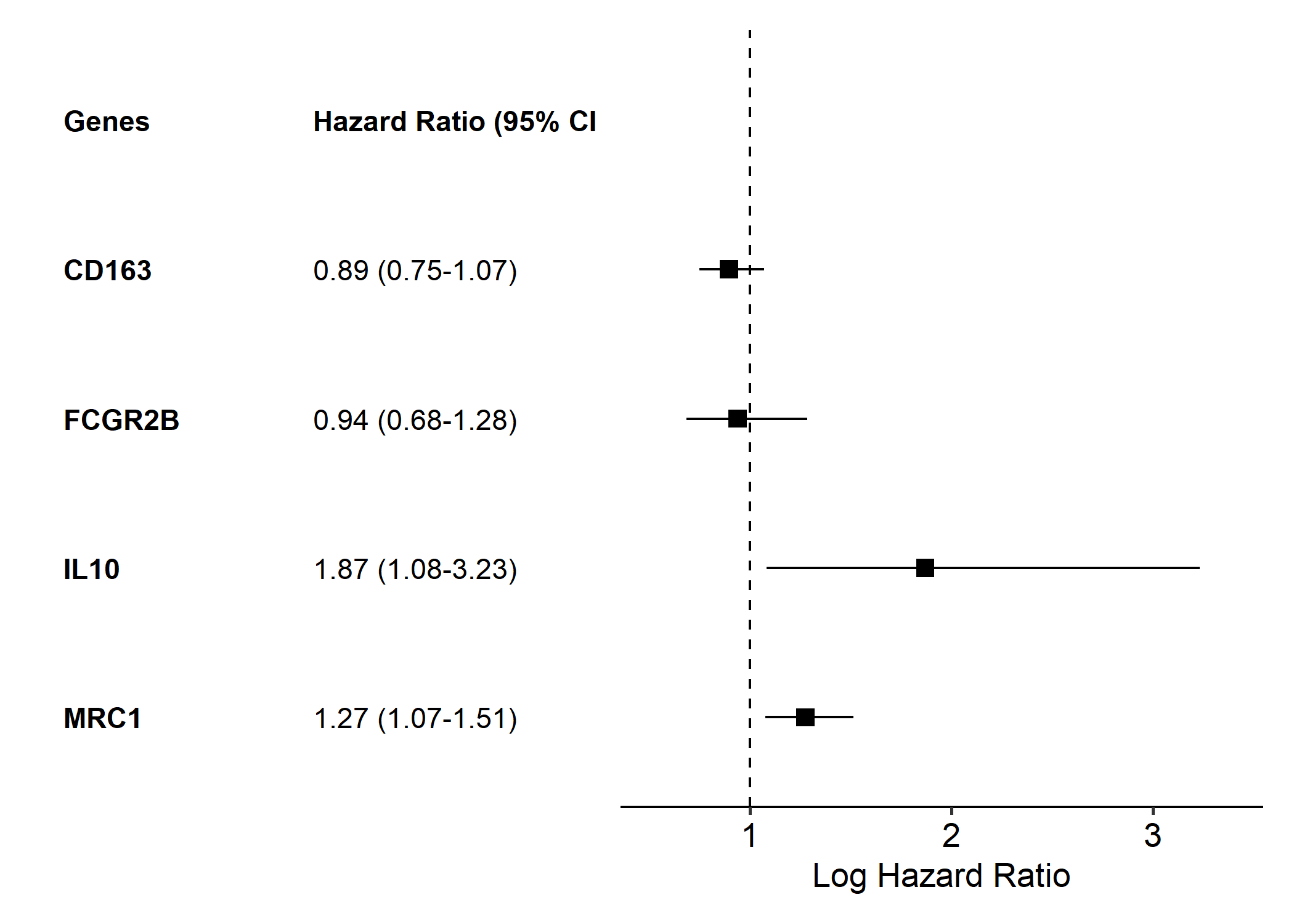

Supplement: Supplementary file 3 — Supplementary Material 3 [file 41598_2025_3557_MOESM3_ESM.tiff]
